# Ciprofloxacin-Modified Degradable Hybrid Polyurethane-Polylactide Porous Scaffolds Developed for Potential Use as an Antibacterial Scaffold for Regeneration of Skin

**DOI:** 10.3390/polym12010171

**Published:** 2020-01-09

**Authors:** Carayon Iga, Terebieniec Agata, Łapiński Marcin, Filipowicz Natalia, Kucińska-Lipka Justyna

**Affiliations:** 1 Department of Polymers Technology, Faculty of Chemistry, Gdansk University of Technology, Narutowicza 11/12, 80-233 Gdansk, Poland; juskucin@pg.gda.pl; 2 Department of Molecular Biotechnology and Microbiology, Faculty of Chemistry, Gdansk University of Technology, Narutowicza 11/12, 80-233 Gdansk, Poland; agata.terebieniec@pg.edu.pl (T.A.); natfilip@student.pg.edu.pl (F.N.); 3 Department of Solid State Physics, Faculty of Applied Physics and Mathematics, Gdansk University of Technology, Narutowicza 11/12, 80-233 Gdansk, Poland; marcin.lapinski@pg.edu.pl

**Keywords:** polyurethane, polylactide, tissue engineering, skin scaffold, antibacterial, degradable, medical

## Abstract

The aim of the performed study was to fabricate an antibacterial and degradable scaffold that may be used in the field of skin regeneration. To reach the degradation criterion for the biocompatible polyurethane (PUR), obtained by using amorphous α,ω-dihydroxy(ethylene-butylene adipate) macrodiol (PEBA), was used and processed with so-called “fast-degradable” polymer polylactide (PLA) (5 or 10 wt %). To meet the antibacterial requirement obtained, hybrid PUR-PLA scaffolds (HPPS) were modified with ciprofloxacin (Cipro) (2 or 5 wt %) and the fluoroquinolone antibiotic inhibiting growth of bacteria, such as *Pseudomonas aeruginosa*, *Escherichia coli*, and *Staphylococcus aureus*, which are the main causes of wound infections. Performed studies showed that Cipro-modified HPPS, obtained by using 5% of PLA, possess suitable mechanical characteristics, morphology, degradation rates, and demanded antimicrobial properties to be further developed as potential scaffolds for skin tissue engineering.

## 1. Introduction

Skin injuries, wounds, burns, and damages of epidermis take place for a variety of reasons, such as contact with hot water, excessive exposition to the sun, different chemicals, or flames, or they can be the result of certain skin diseases [1,2]. Untreated wounds, burns, and injuries may end with a bacterial infection and even death in the worst case. If the epidermis damage is large application of the wound dressing may not be sufficient enough for natural skin regeneration. Thus, functional skin tissue scaffolds (STSs) are being developed to treat large and deep skin defects [1,2]. Fabrication of STS is one of tissue engineering (TE) tasks. TE deals with the fabrication of biologic substitutes that restore, maintain, and improve tissue functions following damage either by disease or traumatic processes. The general principles of TE involve combining living cells with natural or synthetic scaffold to build a three-dimensional (3D) living construct, which is functionally, structurally, and mechanically equal to (or better than) the tissue that is to be replaced [3]. The development of such implantable construct requires careful selection of the biomaterial used for scaffold fabrication. The tissue scaffold should meet strict requirements and act as the extracellular matrix (ECM), which surrounds cells in the body and supports cell proliferation [4].

Biomaterials of polymer origin are employed in the tissue scaffold manufacturing to replace various tissues and organs [5,6,7]. Polymeric materials play a key role in the studies for skin tissue regeneration. The most commonly used synthetic polymers in this field are biocompatible polyurethane (PUR) [8,9,10], polylactide (PLA) [11], polycaprolactone (PCL) [12,13], and ply(glicolide) (PGA) [14].

The versatile synthesis of PUR provides biocompatible, antithrombogenic, and biodegradable materials [15], which are used in a huge variety of medical devices, including endotracheal tubes, vascular grafts, elements of artificial hearts, membranes for dialysis, adhesives for bone regeneration, and materials for dental recovering [16,17,18]. PUR is one of the most popular biomaterials applied for controlled and targeted delivery of drugs in medical devices [19].

Due to the unique segmented structure of PUR, its properties can be modified according to the selected requirements [15], Including the biodegradation profile. According to the literature, PUR usually needs over 6 months to lose 30% of its initial mass in vivo, which is suitable, according to the tissue regeneration requirements [20,21]. It was reported that degradation of scaffold is controlled in such a way that its physicochemical and mechanical properties are maintained for at least 3 to 6 months. Between 1st and 3rd month cells are constantly proliferating, and between 3rd and 6th month regeneration takes place in situ. Henceforth, the scaffold matrix may start losing its mechanical properties and should be metabolized by the body without foreign body reaction approximately between 12 and 18 months [22,23,24,25]. It is worth mentioning here that the degradation products of PUR have to be nontoxic and catalyzable in natural life cycles [26]. The degradation rate may be controlled at different levels. For example, by the application of “fast-degradable” materials like PLA or PEG [27,28], blending with PUR significantly improves their degradation rate. Montini-Ballarin et al. [27] observed that electro spun PUR grafts were losing only 63% of mass after 34 weeks in hydrolytic degradation performed in PBS, while PUR blends with Poly(L-lactic acid) (PLLA) were degrading faster when we blended PUR/PLLA = 50/50 and PUR/PLLA = 10/90, which lost 74 and 90% of their initial mass, respectively, after 8 weeks of study.

Poly(L-lactic acid) (PLLA) is FDA approved biodegradable polyester, commonly used in biomedical applications, such as drug delivery systems, tissue engineering, and biomedical devices that exhibit semi-crystalline structures [29,30,31]. PLLA possesses a high elastic modulus required to withstand high pressure and flow without collapse or degradation until tissue develops and matures in vivo. PLLA has a mechanical response similar to collagen [31]. The degradation process of PLA occurs by hydrolysis and leads to a decrease in the macromolecules of the average length when water reacts with the ester unions present in the polymeric chains [32,33,34]. On the other hand, PUR, which consists of alternating soft segments (SSs) and hard segments (HSs), can also be designed as biodegradable materials [35]. SSs are usually engaged to introduce chemical bonds susceptible to degradation and therefore alternate the material degradation rate. On the contrary, HSs are often degraded through enzymatic mechanisms [35]. Although degradation mechanisms depend on both the PUR soft and hard segments, there are certain mechanisms common to the majority of biodegradable/bioresorbable PUR [27,28]. The composition of the prepolymer or macrodiol has shown to control the in vitro degradation rate of PUR [36]. When soft segments are composed of ester units, the degradation process is mainly the same as the one mentioned before for PLA. In addition, it was observed that PUR with amorphous SSs degrade faster than others with semi-crystalline SSs. Additional degradation of urethane and urea units to free polyamines could take place, depending on the diisocyanate used [36].

Infections related to the biomaterials are often observed with artificial implants and, in many cases, result in the failure of the devices [36]. Various substances known as toxins, proteases, and pro-inflammatory molecules may cause an excessive and prolonged inflammatory response of the host tissues by the bacterial colonization and subsequent infection [37,38]. This can seriously interfere with the wound healing process [39]. Thus, the large focus is to design a skin tissue scaffold that is intrinsically infection-resistant [40]. Ideal antimicrobial skin tissue scaffold should represent certain features, such as the provision of a moist environment to enhance healing [41], providing a broad-spectrum of antimicrobial activity (bacterial growth inhibition) [42], effective absorption of the wound exudates [43], ensuring suitable wound humidity [44], enabling formation of new tissue with no scars [45], and permeability for gases and delivery of nutrients [46]. Therefore, the proper care of skin wounds, burns, and injuries is important for the prevention of microbial infections and trans-epidermal water loss, which lead to accelerated wound regeneration [47]. Thus, restoration of the skin barrier is crucially important for the treatment of injuries. To meet the requirements of antibacterial skin scaffold, the templates are modified with antibiotics, e.g., coming from the fluoroquinolones group [48].

Fluoroquinolones are well-established broad spectrum antibiotics [49,50] with potent bactericidal activity against most common pathogens, which are prevalent at the wound site, such as S. aureus, P. aeruginosa, and E. Coli etc. [51]. Performed microbiological studies have revealed that ciprofloxacin is a relevant antimicrobial agent and works against bacterial species, such as E. coli and *S. aureus* [48,52], which are the main species responsible for wound infections, according to the references. Among them, Ciprofloxacin is one of the most widely used fluoroquinolones for treating a variety of bacterial infections. Its low minimal inhibitory concentration for both Gram-positive and Gram-negative bacteria causes wound infections and the frequency of spontaneous resistance to ciprofloxacin is very low [53].

In this paper, we described the fabrication process of hybrid PUR-PLA scaffolds, containing 5 or 10 wt % of PLA, to improve the scaffold degradability. PUR used in this study was synthesized by the use of amorphous polyester α,ω-dihydroxy(ethylene-butylene adipate) (PEBA) and aliphatic diisocyanate (1,6-hexamethylene diisocyanate) (HDI) [8], according to the references reporting better degradability of PUR containing amorphous macrodiols [27,28] and non-toxic degradation products of PURs obtained by using aliphatic diisocyanates [36,54]. This PUR was characterized in our previous work and recognized as biocompatible [8]. Obtained hybrid PUR-PLA scaffolds (HPPS) were modified with ciprofloxacin (Cipro), a fluoroquinolone antibiotic, which has an inhibitory effect on *S. aureus* growth, which is one of the bacterial species responsible for most common wound infections (37% of all species isolated from the wounds was *S. aureus*) [55]. Performed and described in this article studies of HPPS scaffolds, containing 5% of PLA and modified with Cipro, revealed the suitable mechanical characteristics, morphology, degradation rate, and demanded antimicrobial properties to be further developed as potential scaffolds for skin tissue engineering.

## 2. Materials

All of the salts, solvents, and materials were used without further processing: polylactide (PLA, *M*_w_ = 2000, Sigma Aldrich, Poland) and polyurethane (PUR) was synthesized in our laboratory by Kucińska-Lipka et al. [8]; Dmiethylsulfoxide (DMSO, Sigma Aldrich, Poland), sodium chloride (NaCl, POCH, Poland), hydrochloric acid (HCl, POCH, Poland), cobalt (II) chloride (CoCl_2_, Sigma Aldrich, Poznań, Poland), hydrogen peroxide (H_2_O_2_, POCH, Poland), gelatin (Sigma Aldrich, Poznań, Poland), Ciprofloxacin (Cipro, Sigma Aldrich, Poznań, Poland).

## 3. Methods

### 3.1. Fabrication of Porous Hybrid Polyurethane-Polyester Porous Scaffolds (HPPS)

The fabrication procedure of HPPS was similar to that described in our previous paper [10]. PUR, reported by Kucińska-Lipka et al. [8], was dissolved in dimethylsulfoxide (DMSO) at 20 wt % concentration. PLA was dissolved in DMSO at the same concentration (20 wt %). PLA solution was then mixed with PUR solution at a concentration of 5 or 10 wt % (per mass of PUR). A solution of the PUR-PLA mixture was mixed with the use of a magnetic stirrer at 60 °C for 24 h. Sodium chloride (NaCl, POCH, Poland) at a crystal size in the range 0.6–0.4 µm was then added to the PUR solution until complete solution saturation occurred (high viscosity of mixture). A formulated PUR-PLA-salt mixture was transferred between the flat stainless steel molds and pressed at a hydraulic press (ZUP Nysa) for 3 min at 4.9 MPa pressure (at 20 °C) to reach uniform distribution of the mixture at the molds. Molds were placed at the refrigerator, set at −20 °C overnight to direct the solvent crystallization [56,57,58]. HPPSs were removed from the mold and immersed in warm (40–50 °C) bidistilled water, where, for 7 days, the solvent and the sodium particles were washed out. Water was changed twice a day. Finally, samples of HPPS were dried at 50 °C for 24 h and used for modification and testing. Symbols of samples were given in Table 1.

### 3.2. Modification of HPPS with an Antibacterial Agent from the Group of Fluoroquinolones

The antibacterial factor, ciprofloxacin (Cipro), approved by the FDA, was used in this study in hydrochloride form. The HPPS modification was as follows: Gelatin solution (5 wt %) in DMSO containing 2 or 5 wt % of Cipro was prepared. Gelatin was used for two reasons: One was to increase the solution viscosity and the other was to improve the biocompatibility after implementation of HPPS. HPPS were cut into samples of 40 mm^3^ volume. Samples were placed in the 24-well culturing plates (Bionovo, Legnica, Poland), immersed in 3 mL of Cipro-gelatin solutions containing 2 or 5 wt % of Cipro, and left for 24 h under vacuum at 20 °C to fully penetrate the HPPS. Cipro-modified HPPS were then dried overnight in a laboratory drier set at 60 °C and used for examination. Symbols of samples are given in Table 1.

### 3.3. Samples, Symbols, and Ratios

Table 1 shows symbols and ratios of obtained samples with a brief explanation.

### 3.4. Fourier Transform Infrared Spectroscopy (FTIR)

The FTIR analysis was performed with the use of a Nicolet 8700 Spectrometer in a spectral range from 4000 to 500 cm^−1^, averaging 256 scans with a resolution of 4 cm^−1^.

### 3.5. Mechanical Properties

Tensile strength (*T*_Sb_) and elongation at break (eb) were studied by using the universal testing machine, Zwick & Roell Z020, according to PN-EN ISO 527-2:2012, with a crosshead speed of 100 mm/min and a measuring path of 60.35.

### 3.6. Optical Microscopy

Unmodified and Cipro-modified HPPS were studied by a Digital Microscope U800X at 800× magnification. Optical microscopy (OM) studies were performed before and after short-term degradation studies with selected media. Initial morphological characterization was done by using program ImageJ^®^ software (US National Institute of Health, Bethesda, MA, USA).

### 3.7. Scanning Electron Microscopy (SEM)

SEM of unmodified and Cipro-modified HPPS was performed by using FEI Quanta 250 FEG at an accelerating voltage of 10 kV. Samples were covered with 15 nm layer of gold in sputter-coater Leica EM SCD 500. SEM images were analyzed by ImageJ^®^ software (US National Institute of Health, Bethesda, MA, USA) to calculate the porosity of obtained scaffolds. Energy-dispersive X-ray (EDX) spectroscopy was performed to study an elemental analysis and the chemical composition of unmodified and Cipro-modified HPPSs.

### 3.8. Short-Term Degradation Studies in Selected Media

The short-term degradation studies of obtained unmodified and Cipro-modified HPPSs were performed in selected media: 2 N HCl, 5 M NaOH, and 0.1 M CoCl_2_ in 20% H_2_O_2_. This is a standard procedure previously reported in the literature [59,60]. PUR was cut into round samples of 0.5 cm^2^ area. Prepared samples were dried and weighed in thermobalance (RADWAG MAX50/SX), set at 60 °C. Six samples of each studied PUR materials were then placed in 24-well cell culture plates filled with selected media: oxidative solution of 0.1 M CoCl_2_/20% H_2_O_2_; acidic solution of 2 N HCl or basic solution of 5 M NaOH. Samples were incubated in selected media at 37 °C. Mass changes of samples were examined after 15 days for oxidative, acidic, and basic media. Sample mass change measurements were as follows: Samples were taken out from the container and put into a paper sheet to reduce the medium excess. Samples were then placed in the thermobalance (set at 60 °C), where they were dried to a constant mass and weighed. Mass loss was calculated by Formula 1. The results were the arithmetic mean of six measurements.

(1) $$S=\left(\frac{m_{i}-m_{0}}{m_{0}}\right)\cdot 100\%,$$

where *m_i_* is the sample weight after 1, 3, 7, and 14 days and 1, 2, 3, and 6 months of incubation (g) and *m*_0_ is the sample weight before the test (g).

### 3.9. Microbiological Tests

Antibacterial activity of unmodified and Cipro-modified HPPS was tested by using three bacterial strains belonging to the following species: *Escherichia coli* (Gram negative), *Staphylococcus aureus* (Gram positive), and *Pseudomonas aeruginosa* (Gram negative), respectively, which are potentially Cipro-sensitive bacterial species. The bacterial strains were obtained from a collection of the Department of Molecular Biotechnology and Microbiology, Gdańsk University of Technology, Gdansk, Poland.

All bacterial strains were cultivated in 20 mL of fresh and sterile luria broth (LB) medium. The LB medium contained g/L: casein peptone 10.0; yeast extract 5.0; NaCl 10.0 dissolved in deionized water. Cultivations were carried out in 200 mL sterile Erlenmeyer flasks on a rotary shaker at 170 rpm at 37 °C for 18–24 h. After the incubation time, 100 µL of each bacterial strain culture was transferred into 10 mL of sterile LB medium in 100 mL sterile Erlenmeyer flasks. Next, bacterial strains cultivations were carried out on a rotary shaker at 170 rpm at 37 °C to get the log phase of bacterial growth (OD_600_ values 0.4–0.7). For determination of antibacterial activities, 100 µL of each bacterial strain suspensions in the log phase of growth were placed on sterile LA medium with a sterile glass rod. The LA medium contained g/L: casein peptone 10.0; yeast extract 5.0; NaCl 10.0; agar 15.0. Prior to the examination, unmodified and Cipro-modified HPPS were sterilized by the exposition to UV radiation for 30 min and placed on plates with sterile tweezers. Sterile samples of unmodified and Cipro-modified HPPS scaffolds were placed on bacterial cultures on LA plates and incubated at 37 °C for 24 h. After the incubation, the diameter of the presence or absence of growth inhibition zones around samples of unmodified and Cipro-modified HPPS was measured. All analyses were done in triplicate.

## 4. Results

### 4.1. Fourier-Transform Infrared Spectroscopy

**Figure 1:**
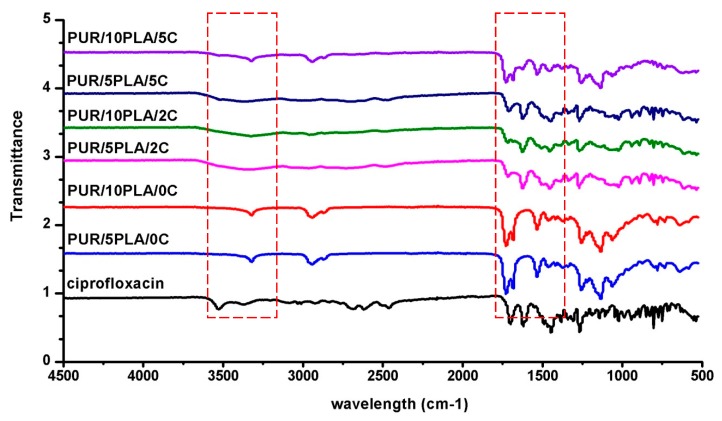
The FTIR spectra of ciprofloxacin used for HPPS modification and FTIR spectra of unmodified or Cipro-modified (2 or 5 wt %) HPPSs, obtained by using 5 or 10 wt % of PLA.

Figure 1 shows the FTIR spectra of ciprofloaxin used for HPPS modification and FTIR spectra of unmodified and Cipro-modified (2 or 5 wt %) HPPSs, which were obtained by using 5 or 10 wt % of PLA.

To analyze the spectra of ciprofloaxin and unmodified and Cipro-modified HPPS (Figure 1), the book of Silverstein et al. [61] and the scientific paper of Tan et al. [61] and Yilgor et al. [62] were used.

The narrow peak detected in case of unmodified HPPS (PUR/10PLA/0C and PUR/5PLA/0C) (Figure 1) at 3328 cm^−1^ corresponded to the stretching of the NH group present in urethane linkages. Bands observed at 2941 and 2864 cm^−1^ indicated stretching of aliphatic asymmetric and symmetric CH_3_ and CH_2_ groups present in HPPS, coming from PUR components (macrodiol and diisocyanate) and PLA chemical structure. A total of 1725 cm^−1^ was observed stretching the carbonyl groups present in the PLA structure. A further band was indicated at 1681 cm^−1^ related to the presence of urethane linkages in obtained HPPS. The confirmation of the presence of urethane linkage in the HPPS structure was the band observed at 1522 cm^−1^ concerning stretching of C–N. At 1466 and 1373 cm^−1^ bands were observed indicating the deformation of CH_3_ and CH_2_ groups of HPPS. Between 1262 and 1053 cm^−1^ was recognized the stretching of –C(O)–O– and C–O– of HPPS, coming mainly from PUR macrodiol and PLA structures. Between 778 and 586 cm^−1^ indicated out of plane deformation of CH_3_, CH_2_, NH, and OH.

In terms of Cipro-modified HPPS with 2 or 5 wt % of ciprofloxacin (Figure 1) (PUR/10PLA/5C, PUR/5PLA/5C, PUR/10PLA/2C, and PUR/5PLA/2C), the arrangement of the bands was similar to those observed for unmodified HPPS and ciprofloxacin: Between 3667–3123 cm^−1^ was identified in the stretching of the COOH group present in the ciprofloxacin and stretching of NH groups of both ciprofloxacin and in the HPPS structure. In the range of 3116–2886 cm^−1^ the stretching of aromatic and cycloaliphatic rings present in the structure of ciprofloxacin was noted, and the asymmetric and symmetric stretching of aliphatic CH_3_ and CH_2_ groups present in HPPS (macrodiol and diisocyanate of PUR and in the PLA structure) was noted. Between 2721–2479 cm^−1^ was noted the stretching of double bonds present in the aromatic ring. A total of 1714 cm^−1^ indicated the stretching of carbonyl groups of PLA. At 1620 cm^−1^ was observed the band described as stretching of the urethane linkages and stretching of C–N, confirming the presence of urethane linkage in HPPS structure. At 1449 cm^−1^ was observed the stretching of the rings present in ciprofloxacin. Between 1383 and 1268 cm^−1^, aromatic ring overtones related to the aromatic ring substitution was observed. Between 1169 and 1015 cm^−1^, the stretching of –C(O)–O– and –C–O– was observed. Between 938–520 cm^−1^, out of plane deformation of CH_3_, CH_2_ NH, and OH was indicated.

### 4.2. Mechanical Properties

**Figure 2:**
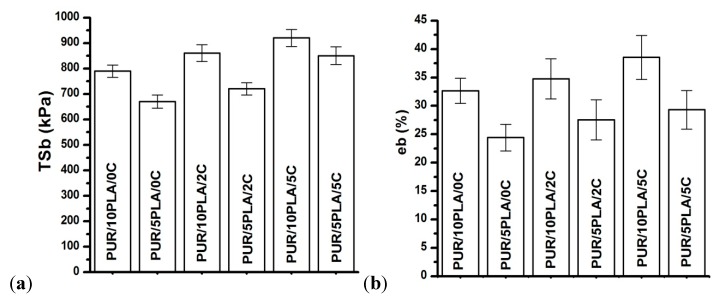
Tensile strength (**a**) and elongation at the break (**b**) of the unmodified and Cipro-modified HPPS.

Figure 2 showed tensile strength (*T*_Sb_) and elongation at the break (e_b_) of the obtained unmodified and Cipro-modified (2 or 5 wt %) HPPS, containing 5 or 10 wt % of PLA.

*T*_Sb_ of PUR/5PLA/0C (Figure 2a) was 670 ± 26 kPa, and eb (Figure 2b) was 24 ± 2%. The HPPS modification with ciprofloxacin significantly increased the T_Sb_ of the obtained Cipro-modified HPPS (PUR/5PLA/2C = 720 ± 24 kPa, PUR/5PLA/5C = 850 ± 34 kPa) and slightly increased the eb (PUR/5PLA/2C = 28 ± 4%, PUR/5PLA/5C = 30 ± 3%). The T_Sb_ of PUR/10PLA/0C was 790 ± 24 kPa, and eb was 32 ± 2%. Application of ciprofloxacin modification in HPPS caused a large improvement of *T*_Sb_ (PUR/10PLA/2C = 860 ± 33 kPa and for PUR/10PLA/5C = 920 ± 33 kPa) as it was observed in case of HPPS containing 5 wt% of PLA. The eb increased slightly (PUR/10PLA/2C = 34 ± 5%, PUR/10PLA/5C = 39 ± 4%). The HPPS, which contained 5 wt % of PLA had lower *T*_Sb_ than HPPS, containing 10 wt % of PLA, but in the case of eb, no significant improvement was noted.

### 4.3. Scanning Electron Microscopy

**Figure 3:**
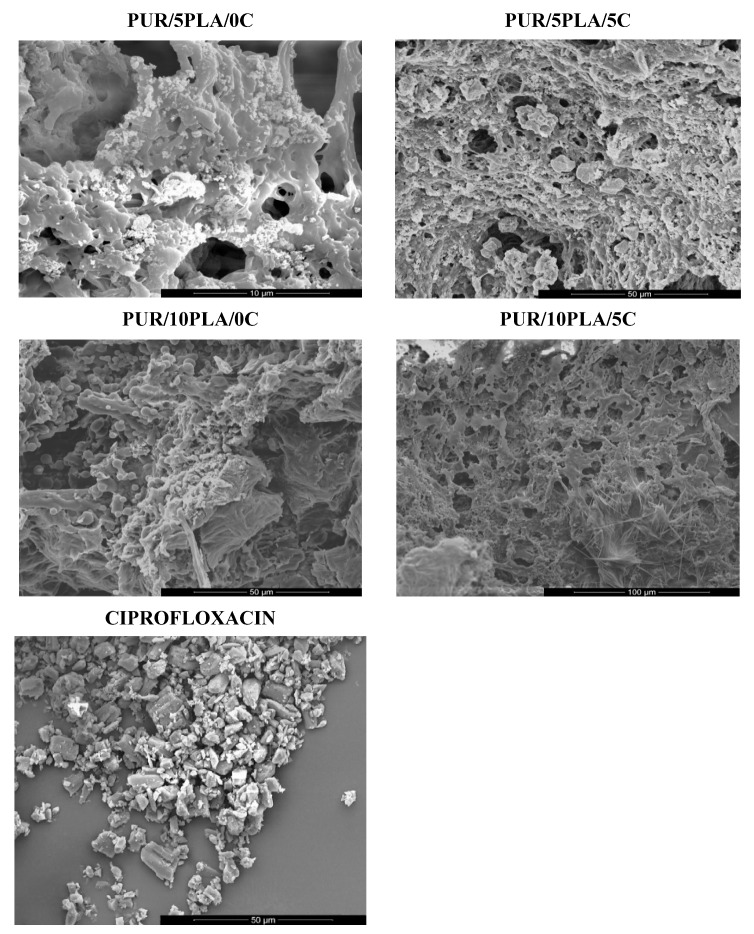
SEM images of unmodified and Cipro-modified (2 or 5 wt %) HPPS, obtained by using 5 or 10 wt % of PLA and a SEM image of ciprofloxacin used for HPPS modification.

**Figure 4:**
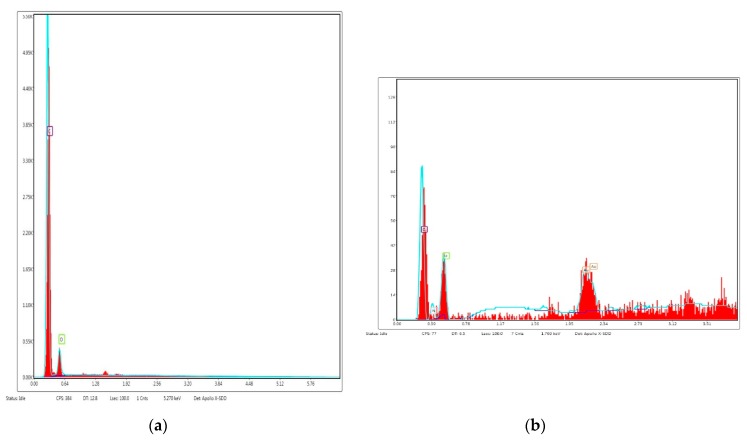
Selected energy-dispersive X-ray (EDX) spectra of (**a**) PUR/5PLA/0C and (**b**) PUR/5PLA/5C.

**Figure 5:**
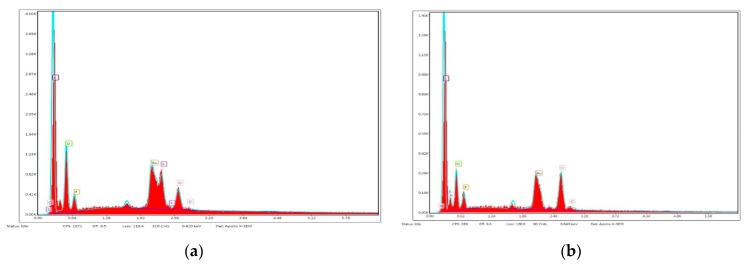
Selected EDX spectra of (**a**) PUR/10PLA/0C and (**b**) PUR/10PLA/5C.

**Figure 6:**
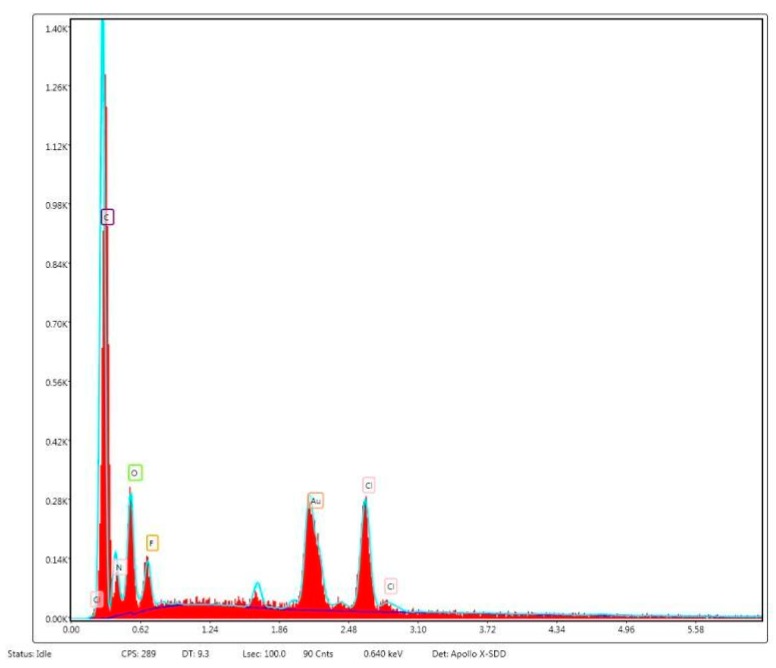
Selected EDX spectra of ciprofloxacin.

Figure 3 shows SEM images of unmodified and Cipro-modified (2 or 5 wt %) HPPS obtained by using 5 or 10 wt % of PLA. Figure 3 presents the image of ciprofloxacin used for HPPS modification. Figure 4 and Figure 5 shows the results of EDX analysis performed during SEM studies of unmodified and Cipro-modified HPPS. Figure 6 shows the EDX spectra of ciprofloxacin.

SEM images (Figure 3) confirmed the porous structure of unmodified and Cipro-modified HPPS. Porosity of HPPS is given in Table 2.

In case of HPPS obtained by using 5 wt % of PLA, the homogenous porous structure (86%) was observed (Figure 3 and Table 2) in pore sizes in the range of 50–375 µm. Pores were interconnected, which is favorable in case of porous materials dedicated to the tissue engineering. Modification with ciprofloxacin (Figure 3 and Table 2) did not cause significant changes in the porosity of HPPS containing 5 wt % of PLA (PUR/5PLA/2C = 87% and PUR/5PLA/5C = 85%) or on the pore sizes (47–320 µm for PUR/5PLA/2C and 32–297 µm for PUR/5PLA/5C).

For HPPS obtained by using 10 wt % of PLA, a high % of porosity (84%) was observed as well (Figure 3 and Table 2). The pores were interconnected and the sizes were between 67–332 µm. Ciprofloxacin modification (Figure 3) caused significant changes in the HPPS morphology. A large decrease of porosity was noted (Figure 3 and Table 2) (up to 72% for PUR/10PLA/2C and up to 64% for PUR/10PLA/5C), and decrease of pore sizes (or even their full closure) was observed, which was increasing with the ciprofloxacin amount.

The EDX analysis (Figure 4 and Figure 5) of unmodified and Cipro-modified HPPS confirmed the presence of chemical elements of PUR and PLA structures: carbon, oxygen, and nitrogen. In case of Cipro-modified HPPS, the EDX analysis revealed the presence of ciprofloxacin (Figure 6). The EDX spectra identified the presence of elements like chloride and fluorine characteristics for ciprofloxacin hydrochloride salt used in the study. Presence of gold at the EDX spectra was related to the sputter coating of the HPPS samples prior to the SEM study.

### 4.4. Short-Term Interaction with Selected Media

**Figure 7:**
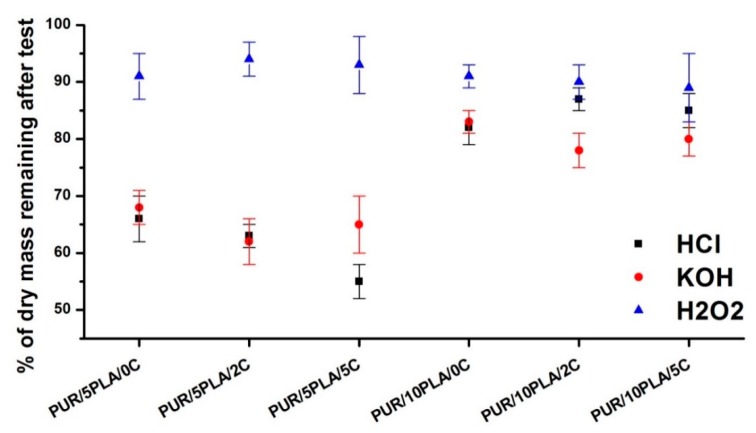
Dry residue (%) of unmodified and Cipro-modified HPPS, obtained by using 5 or 10 wt % of PLA after 15 days of incubation in selected media: 2 N HCl, 5M KOH, and 0.1 M CoCl_2_ in H_2_O_2__._

Figure 7 showed the % of dry mass remaining after the test of short-term interactions performed with unmodified and Cipro-modified HPPS containing different amounts of PLA. Samples were studied after 15 days of incubation in selected media: 2 N HCl, 5M KOH, and 0.1 M CoCl_2_ in 20% H_2_O_2_.

Figure 7 shows that HPPS obtained by using 10 wt % of PLA were less sensitive in the selected environments than those obtained by using 5 wt % of PLA. For unmodified HPPS containing 10 wt % of PLA, the dry residue was 82 ± 3% in the acidic environment, 83 ± 2% in the basic environment, and 91 ± 2% in the oxidative environment. It shows that 18% and 17% of HPPS containing 10 wt % of PLA degraded in acidic and basic environment, respectively, and 9% degraded in the oxidative environment. The ciprofloxacin modification (both 2 and 5 wt %) of HPPS, containing 10 wt % of PLA, did not cause significant mass changes. In the acidic environment, the mass decrease was an average of 13% and 15%, respectively, when 2 and 5 wt % of ciprofloxacin was added. In the basic environment, the mass decrease was 22% and 20% with an increase of the ciprofloxacin amount from 2 to 5 wt %, respectively. In the oxidative environment, the mass decrease was 10% and 11% for 2 and 5 wt % of ciprofloxacin, added respectively.

In case of unmodified HPPS obtained with 5 wt % of PLA, the mass decrease was about 15% higher in comparison to the unmodified HPPS obtained with the use of 10 wt % of PLA. Respectively, it was as follows: 34% in the acidic environment and 32% in the basic environment. In the oxidative environment, the mass decrease was comparable to the HPPS samples obtained by using 10 wt % of PLA and equal to the 9%. Introduction of ciprofloxacin in case of HPPS obtained with 5 wt % of PLA had a larger influence on the degradation of these materials than in the case of HPPS obtained with 10 wt% of PLA. In the acidic environment, the mass decrease was 43% and 45% for PUR/5PLA/2C and PUR/5PLA/5C, respectively. In the basic environment, the noted mass decrease was 38% and 40% for PUR/5PLA/2C and PUR/5PLA/5C, respectively. In the oxidative environment, the mass decrease was about 10% and was comparable with the mass decrease of Cipro-modified HPPS samples obtained with 10 wt % of PLA.

**Figure 8:**
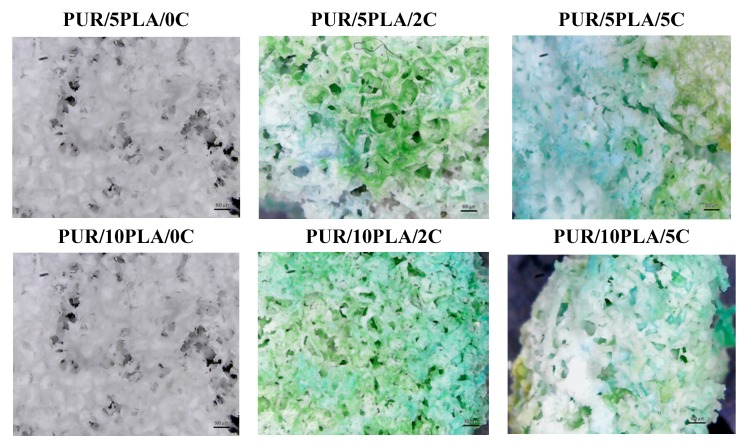
Optical microscopy images of unmodified and Cipro-modified HPPS obtained by using 10 wt % after 15 days of incubation.

Unmodified and Cipro-modified HPPS, which were interacting with the acidic and basic environments after 15 days of incubation and drying to the constant mass (at 60 °C), were characterized by high fragility, which made it impossible to use in optical microscopy studies. Such changes were not observed in case of materials after oxidative degradation, which were stable and didn’t lost large % of mass. Optical microscopy images before and after short-term interactions with the oxidative environment study was presented in Figure 8. The blue-green color of samples came from anhydrous cobalt chloride.

### 4.5. Microbiological Tests

**Figure 9:**
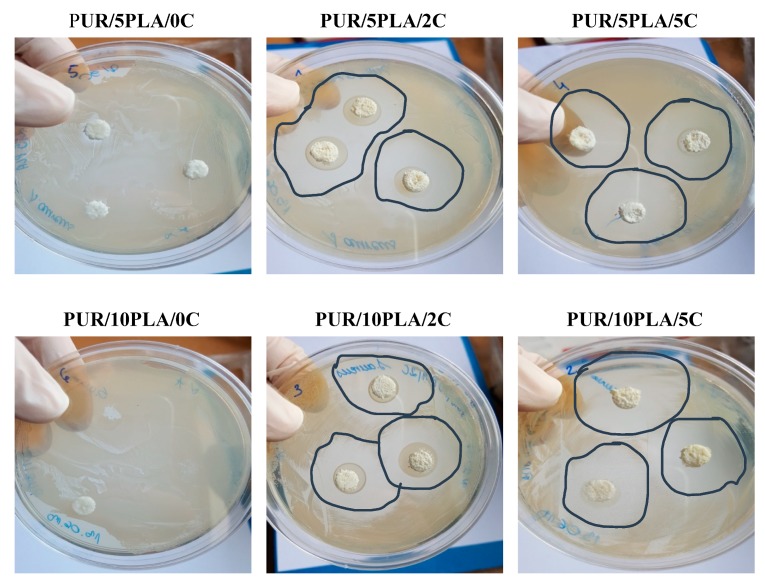
The effect of antimicrobial activity of Cipro-modified HPPS (2 or 5 wt % of ciprofloxacin) against *S. aureus*.

Performed microbiological tests (Figure 9) revealed the presence of inhibition zones (Table 3) of *S. aureus* growth when HPPS was modified with ciprofloxacin (2 and 5 wt %). The diameters of inhibition zones increased with the amount of ciprofloxacin added to the HPPS. What had to be marked is the formation of uneven inhibition zones. This could be related to the porous structure of HPPSs, their different composition (% of PLA and % Cipro), and the distribution of both PLA and Cipro in the porous structure of HPPS. This test determined that such Cipro-modification of porous structures evoke antimicrobial activity, which can be used for tissue engineering purpose. There were no growth inhibition zones for *E. coli* and *P. aeruginosa*. *P. aeruginosa* was frequently developed as resistant against drugs. Although ciprofloxacin is a commonly used antibiotic for *P. aeruginosa*, there are available reports, which indicate that even 30%–37% of *P. aeruginosa* isolates are ciprofloxacin-resistant, whereas *E. coli* strain resistance represented approximately 11% [55,63]. Figure 8 shows the effect of antimicrobial activity of Cipro-modified HPPS (2 or 5 wt % of ciprofloxacin) against *S. aureus* in comparison to the unmodified HPPS serving as the control.

## 5. Discussion

In this study, the fabrication process of hybrid PUR-PLA scaffolds (HPPS) was described. These HPPSs contained 5 or 10 wt % of PLA, selected as one of the “fast-degradable” polymers, which, when admixed with the PUR, were proven to improve its degradation rate [27,28]. Moreover, biocompatible PUR used in this study [8,10] was synthesized with amorphous macrodiol PEBA [8], which, according to the references, may improve the degradation profile [39] of such HPPSs. Obtained HPPSs were modified with ciprofloxacin to improve the antibacterial effects of HPPSs dedicated for skin regeneration. Ciprofloxacin is a fluoroquinolone antibiotic inhibiting *S. aureus* growth, which is one of the bacterial species responsible for the most common wound infections [40,46,48,50,51,61]. The FTIR analysis of obtained unmodified and Cipro-modified HPPSs revealed the presence of chemical functional groups characterizing PURs (urethane linkages), PLA (ester linkages) [62], and ciprofloxacin (complex structure) bonded to the HPPS. The EDX analysis confirmed the presence of ciprofloxacin in the HPPS systems, which was in good agreement with FTIR studies.

Performed FTIR spectroscopy showed that, in case of unmodified HPPS, the FTIR band intensity grew with the amount of PLA added. The same tendency was noted for Cipro-modified HPPS samples, where the band intensity improved with the increase of the ciprofloxacin amount in the HPPS sample. This may suggest that the formation of additional hydrogen bonds, over those present in the native PUR structure, reinforce the structure of obtained HPPSs [62]. The presence of hydrogen bonds, which increased with the amount of PLA and ciprofloxacin added, could be an explanation for mechanical properties of obtained HPPSs. Scaffolds containing 10 wt % of PLA revealed largely higher *T*_Sb_ (670 ± 26 kPa) than those obtained with 5 wt % of PLA (790 ± 24 kPa). The application of ciprofloxacin additionally increased the *T*_Sb_ value of both HPPS containing 5 wt % (PUR/5PLA/2C = 720 ± 24 kPa, PUR/5PLA/5C = 850 ± 34 kPa) and 10 wt % of PLA (PUR/10PLA/2C = 860 ± 33 kPa, PUR/10PLA/5C = 920 ± 33 kPa). A higher amount of hydrogen bonds caused physical crosslinking of the HPPS structure [64]. What needs to be underlined is that, from the mechanical point of view, both materials (except PUR/10PLA/5C) met the criteria for skin regeneration. The tensile strength of the skin covering the area of the forearm and face was reported to be between 200–850 kPa [65], depending on the skin composition, and the mean failure strain was 25.45 ± 5.07% [66]. From a morphological point of view, only HPPS obtained by using 5 wt % of PLA represented suitable homogenous and interconnected morphology even after ciprofloxacin modification, which was in contrary to the HPPS obtained with the use of 10 wt % of PLA, where porosity decreased (even complete closure of pores was observed [67]) with the amount of ciprofloxacin added. This is the factor which disqualified HPPS containing 10 wt % of PLA samples for further tissue engineering applications [67]. However, the EDX analysis showed that ciprofloxacin was present in both modified HPPSs containing 5% or 10% of PLA. Thus, the modification with ciprofloxacin of hybrid PUR-PLA scaffolds is possible. In terms of degradation rate, better performance was noted for HPPS containing 5 wt % of PLA in comparison to the HPPS samples containing 10 wt % of PLA. This could be explained by the presence of reinforcing hydrogen bonds [62,64] in the HPPS structure; during HPPS fabrication PLA could precipitate the solution and later on could be enclosed in the PUR matrix. Due to this, PLA particles may act as an inactive filler [64], which causes physical hydrogen bonds and strengthens the HPPS structure. In the point of degradation rate, a better degradation profile was noted for HPPS containing 5 wt % of PLA. The HPPSs containing 10 wt % of PLA were more resistant to the selected media. These data are in good agreement with studies performed by Montini-Ballarin et al. [27,28]. The performed study of antibacterial properties against Cipro-sensitive *S. aureus* strain depended on the amount of ciprofloxacin added to the HPPS, but was not dependent on the % of PLA introduced into the HPPS (See Table 3). The observed uneven inhibition zones could be related to the fact that HPPSs are porous materials and that Cipro could not be homogenously dispersed in its structure. It was revealed that Cipro-modified HPPSs represented antimicrobial activity, thus they can be developed in terms of antimicrobial materials for tissue engineering purposes. Performed studies revealed that the aim of fabricating degradable and antibacterial Cipro-modified HPPSs was achieved. HPPSs obtained by using 5% of PLA and modified with Cipro were selected for further development for skin regeneration. These materials had suitable chemical composition, mechanical properties, degradation profiles, morphology, and antimicrobial activity for the proposed skin tissue scaffold.

## 6. Conclusions

In this study paper, we described the fabrication process of degradable HPPS containing “fast-degradable” polymer in the amount of 5 and 10 wt %. To reach the antibacterial character of HPPSs, the samples were modified with ciprofloxacin. Performed studies confirmed that PLA and ciprofloxacin were present in the chemical structure of obtained HPPSs. Mechanical tests and morphology studies show that more suitable characteristics for skin tissue regeneration possess Cipro-modified HPPSs containing 5 wt % of PLA. These samples represented a better degradation rate in a performed short-term interactions study with selected media: 2 N HCl, 5 M KOH, and 0.1 M CoCl2 in H_2_O_2_. On the other hand, the studies of microbiological tests seem to not to have revealed large differences between Cipro-modified HPPSs containing 5 or 10 wt % of PLA. They represented comparable inhibition zone dimensions, which increased with the amount of ciprofloxacin amount added to the HPPS. Thus, performed studies showed that Cipro-modified HPPSs samples containing 5 wt % of PLA seemed to be suitable to be developed further for the skin tissue scaffold.

## Figures and Tables

**Table 1:** Symbols and ratios of unmodified and Cipro-modified hybrid biocompatible polyurethane- polylactide (PUR-PLA) scaffolds (HPPSs) with a brief explanation.

Symbol	Explanation	Ratios			
		PUR	PLA	CIPRO	GELATIN
**PUR/10PLA/0C**	Unmodified HPPS obtained with 10 wt % of PLA, not modified with Cipro	17	2	0	1
**PUR/10PLA/2C**	HPPS obtained with 10 wt % of PLA, modified with 2 wt % of Cipro	41.5	5	1	2.5
**PUR/10PLA/5C**	HPPS obtained with 10 wt % of PLA, modified with 5 wt % of Cipro	16	2	1	1
**PUR/5PLA/0C**	Unmodified HPPS obtained with 5 wt % of PLA, not modified with Cipro	18	1	0	1
**PUR/5PLA/2C**	HPPS obtained with 5 wt % of PLA, modified with 2 wt % of Cipro	44	2.5	1	2.5
**PUR/5PLA/5C**	HPPS obtained with 5 wt % of PLA, modified with 5 wt % of Cipro	17	1	1	1

**Table 2:** Porosity of unmodified and Cipro-modified HPPS.

Symbol	Porosity (%)
**PUR/5PLA/0C**	86%
**PUR/5PLA/2C**	87%
**PUR/5PLA/5C**	85%
**PUR/10PLA/0C**	84%
**PUR/10PLA/2C**	72%
**PUR/10PLA/5C**	64%

**Table 3:** *S. aureus* inhibition zones detected for unmodified and Cipro-modified HPPS.

Symbol	Inhibition Zone (mm)
**PUR/5PLA/0C**	0
**PUR/5PLA/2C**	15
**PUR/5PLA/5C**	20
**PUR/10PLA/0C**	0
**PUR/10PLA/2C**	16
**PUR/10PLA/5C**	22

## Data Availability

The raw/processed data required to reproduce these findings cannot be shared at this time as the data also forms part of an ongoing study.
